# Evaluation of the Efficacy of the Vaccine Production Process in Removing Residual Host Cell DNA from the Vero Cell Rabies Vaccine

**DOI:** 10.3390/vaccines12121379

**Published:** 2024-12-06

**Authors:** Jia Li, Ruowen Pan, Fengyi Yue, Tie Gao, Xiaohong Wu, Leitai Shi, Yunpeng Wang, Danhua Zhao, Zhaohui Lan, Hongxu Chen, Qiang Ye, Shouchun Cao

**Affiliations:** 1 National Institutes for Food and Drug Control, No. 31, Huatuo Road, Beijing 102629, China; lijiarv@nifdc.org.cn (J.L.); wuxiaohong@nifdc.org.cn (X.W.); taige@nifdc.org.cn (L.S.); wangyunpeng@nifdc.org.cn (Y.W.); zhaodanhua@nifdc.org.cn (D.Z.); qiangyee@nifdc.org.cn (Q.Y.); 2 Hualan Biological Vaccine Inc., Jia No.1-1, Hualan Ave., Xinxiang 453003, China; prw0023@hualan.com (R.P.); yfy3668@hualanbacterin.com (F.Y.); 3 SCIEX China, 5F, Building 1, 24 Yard, Jiuxianqiao Mid Road, Chaoyang District, Beijing 100015, China; tie.gao@sciex.com (T.G.); zhaohui.lan@sciex.com (Z.L.); hongxu.chen@sciex.com (H.C.)

**Keywords:** rabies virus, Vero cells, HCD removal, DNA fragment size distribution

## Abstract

Background: The Vero cell rabies vaccine is currently the most widely used human rabies vaccine. However, owing to the presence of residual host cell DNA (HCD) in the final product and the potential tumorigenicity of the DNA of high-passage Vero cells, the WHO not only sets a limit on the number of times cells used in production can be passaged, but also imposes strict requirements on the amount of residual HCD in the final vaccine product. Objectives: To systematically reduce the HCD level in the final vaccine product, multiple purification steps are included in the vaccine production process. This study investigated the effectiveness of key production steps in antigen recovery and DNA removal. Methods: The residual HCD fragment content and size distribution were detected using fluorescence quantitative PCR (qPCR) and capillary gel electrophoresis (CGE), and the rabies virus glycoprotein antigen content was detected using enzyme-linked immunosorbent assay (ELISA). The antigen recovery rate and HCD removal rate in each key process were calculated to evaluate the scientific basis and effectiveness of each production step. Additionally, the stability of the process was studied using multiple commercial batches of the product. Results: The results revealed that the total antigen recovery rate in the production process described in this report was no less than 8.5%, and the effective removal rate of residual HCD was not lower than 99.99%. Moreover, the amount of residual HCD in the final product was far below the quality standard of 2 ng/dose, and most of the residual HCD fragments were smaller than 200 bp. The results of the process stability studies on multiple commercial batches showed that the bulk human rabies vaccine produced by this process had excellent safety and efficacy and that the production process was stable and thus suitable for large-scale batch production. Conclusions: The production process described in this study achieved effective recovery of viral antigens and efficient removal of residual HCD, and the process was stable and controllable, enabling the continuous and stable production of vaccine products that meet WHO recommendations and the relevant requirements of the current edition of the Chinese Pharmacopeia. In addition, this study provides theoretical guidance for optimizing the vaccine production process.

## 1. Introduction

Rabies virus is a zoonotic infectious virus that affects the nervous system [1]. The mortality rate of rabies virus infection is nearly 100%. Each year, approximately 60,000 people die from rabies worldwide, and most cases occur in Asia and Africa. At present, successful treatment methods for rabies do not exist, and prevention and control of the disease rely solely on vaccination with rabies vaccine and rabies immunoglobin, such as equine rabies immunoglobulin. The current production process for human rabies vaccines generally involves inoculating cells with a fixed strain of the rabies virus, harvesting the infected cells and creating single-cell suspensions, and then preparing the vaccine in bulk through ultrafiltration, inactivation, and gel chromatography purification, followed by dilution to produce the finished vaccine product.

At present, the cells used in the production of human rabies vaccines mainly include passaged Vero cells, hamster kidney primary cells, chicken embryo primary cells, and passaged human diploid cells. Among them, Vero cells are derived from the kidney cells of the African green monkey [2], and a passage cell line is formed after stable passaging. They are widely used in large-scale vaccine production due to their characteristics of rapid growth, easy cultivation, suitability for modern culture methods (such as bioreactors and cell factories), ease of use in scaling up and standardizing vaccine production, and conduciveness to improving vaccine yield [3,4].

However, due to the potential tumorigenicity of high-passage Vero cell DNA and the fact that residual host cell DNA (HCD) cannot be completely removed during the vaccine production process [5,6,7], the World Health Organization (WHO) considers residual HCD a process impurity that lowers product safety and should be removed as much as possible [8]. During the production process, methods such as removal and/or inactivation can be used to reduce the content of residual HCD in the virus passaging, cell culture, production, and purification processes [9].

Currently, various countries have strict requirements for the acceptable amount of residual Vero cell DNA in biological products to ensure the safety of vaccines. The WHO requires that the residual DNA in rabies vaccine be tested at the bulk stage using appropriate methods, with a limit of <10 ng/dose [10]. Additionally, the FDA requires the testing of residual DNA in rabies vaccine at the finished product stage using appropriate methods, with a limit of <10 ng/dose, and also requires the testing of DNA fragment size distribution [11]. The current edition of the Chinese Pharmacopeia requires that the rabies vaccine be tested at the bulk stage, with a limit of ≤3 ng/dose [12].

At present, methods for determining the amount of residual exogenous DNA primarily include DNA probe hybridization, fluorescence staining, and quantitative PCR. In quantitative PCR, the amount of PCR product is detected by incorporating fluorescently labelled specific probes or fluorescent dyes during the PCR reaction. By continuously monitoring the changes in the fluorescence intensity in the reaction system, the changes in the amount of specific amplification products can be instantly reflected. In this method, specific detection probes and primers should be designed based on the host cell type, and corresponding reference materials should be established to prepare the standard curve. Due to its high specificity, high sensitivity, and wide linear range, this method is widely used in the detection of nucleic acids [13]. However, because there is currently no gold-standard method for determining the representative fragments of residual HCD in vaccine products and because different lengths of target amplified fragments can lead to significant differences in quantitative results, the established methods require substantial validation testing. In the current edition of the Chinese Pharmacopeia, this method is used for the detection of residual HCD in bulk human rabies vaccine.

For human rabies vaccines prepared from Vero cells, the WHO’s recommendation is that the limit of HCD residues be no more than 200 bp [14], and that the distribution of residual HCD fragments in the product should be examined to improve vaccine safety evaluation. Capillary gel electrophoresis (CGE) is a technology that uses capillaries as separation channels and a high-voltage direct-current electric field as the driving force to achieve high-resolution separation in the buffer of the gel system based on the difference in the molecular weights of the analytes. It can be widely applied to the analysis of proteins and nucleic acids. Due to its high sensitivity and accuracy, CGE can also be used to detect the size distribution of residual HCD fragments in vaccines.

The preparation process of the Vero cell rabies vaccine is complex. In addition to ensuring the efficacy of the vaccine, the safety of the vaccine should also be ensured by continuously investigating the efficiency of the production process in removing residual HCD. This study investigated the rate of residual HCD removal in each key process during the production of the human rabies vaccine, and evaluated the residual HCD content, size distribution of residual HCD fragments, and antigen content of each intermediate control sample to scientifically evaluate the correlation between effective antigen recovery and efficient removal of residual HCD, thereby ensuring the safety and efficacy of the vaccine product. This study not only provides a scientific basis for rabies vaccine production process research but also offers guidance for the continuous improvement of the entire vaccine production process.

## 2. Materials and Methods

### 2.1. Experimental Materials

The Vero cells (Catalogue no. CCL-81, passage 120) were purchased from the American Type Culture Collection (ATCC) (Manassas, VA, USA). The fixed rabies virus strain CTN-1 V (Catalogue no. CTN-1V_5_,) was obtained from National Institutes for Food and Drug Control (NIFDC) (Beijing, China). The process rabies vaccine control samples were provided by Hualan Biological Vaccine Inc. (Xinxiang, China). The national reference standard for Vero cell DNA quantification (limited to qPCR method) was purchased from the NIFDC. The DNeasy^®^ Blood & Tissue Kit (250) was purchased from QIAGEN (Hilden, Germany). The Vero residual DNA detection kit was purchased from SHENTEK (Huzhou, China). The rabies viral antigen ELISA kit was purchased from the Wuhan Institute of Biological Products (Wuhan, China), and the 7th International Standard for Rabies Vaccine was purchased from the National Institute for Biological Standards and Control (NIBSC) (Herts, UK). DNA-coated capillaries (with an internal diameter of 100 μm) and dsDNA 1000 gel were purchased from SCIEX (Brea, CA, USA); SYBR Gold fluorescent dye and RNase I (10 U/μL) were purchased from Thermo Fisher (Carlsbad, CA, USA). The 10× Tris borate-EDTA (TBE) buffer was purchased from Sigma Aldrich (St. Louis, MO, USA). The DNA marker for capillary electrophoresis was prepared by the NIFDC, with synthesized 100, 200, 500, and 1000 bp DNA fragments, each with an initial concentration of 400 ng/µL, mixed in equal proportions and diluted to 20 ng/µL. For gel buffer preparation, 20 mL of double-deionized water was added to the solid gel and stirred until the solid gel was completely dissolved, then the mixture was diluted 12-fold with 1× TBE buffer to obtain the separation gel buffer. An appropriate amount of gel buffer was mixed with SYBR Gold dye at a volume ratio of 10,000:1 for CGE-LIF analysis.

### 2.2. Preparation of Process Intermediates for Rabies Vaccine

**Figure 1:**
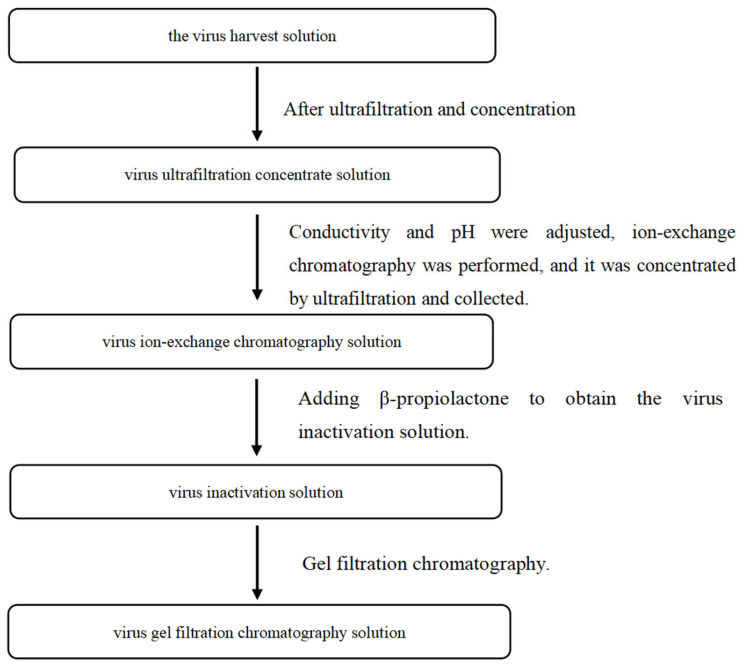
Process steps and samples at different stages of rabies vaccine purification.

Two batches of human rabies vaccine were produced according to the established process, and the intermediates from each batch were collected, including the rabies virus CTN-1V harvest solution, virus ultrafiltration concentrate solution, virus ion-exchange chromatography solution, virus inactivation solution, and virus gel filtration chromatography solution. The process steps are shown in Figure 1.

### 2.3. Determination of Residual DNA Removal Efficiency and Antigen Recovery Rate in Various Steps of Human Rabies Vaccine Production

#### 2.3.1. Determination of Residual HCD via qPCR

After the samples from each process step were appropriately concentrated, the total DNA was extracted using the DNeasy^®^ Blood & Tissue Kit (250). The nucleic acid content was determined by Nanodrop, and the value was expected to be no less than 30 ng/μL. Quantitative PCR was performed according to the third method in the General Rule 3407 of the Chinese Pharmacopeia, Volume IV (2020 Edition), to determine the level of exogenous residual DNA [12]. A Vero cell residual DNA detection kit (PCR-fluorescent probe method) was used, and the Pharmacopeia-recommended detection probe was used, which was a 154 bp fragment from the Vero cell genome-specific 172 bp satellite sequence. The standard was diluted 10-fold to a range of 0.003 pg/µL to 300 pg/µL, and the samples to be tested were diluted within this linear range for detection.

#### 2.3.2. Distribution of Residual HCD Fragments Determined by CGE

The methods for extracting Vero HCD from the samples at each process stage were the same as those described in Section 2.3.1. Before CGE detection, 1 μL of RNase I was added to each sample, followed by incubation at 37 °C for 30 min. A PA 800 Plus pharmaceutical analysis system was used in the CGE method and was equipped with a laser-induced fluorescence detector (the excitation wavelength was 488 nm and the emission wavelength was 520 nm). The capillaries were washed with double-deionized water at 20 psi for 5 min and gel buffer at 20 psi for 5 min. The sample injection condition was 0.2 psi for 2 s; the separation voltage was −9 kV for 20 min, the capillary temperature was 20 °C, and the sample chamber temperature was 10 °C. Between each injection, gel buffer was used to wash the capillaries at 20 psi for 3 min.

#### 2.3.3. Determination of the Rabies Virus Glycoprotein Antigen Content via ELISA

A sandwich ELISA was used to detect the relative content of rabies virus glycoprotein antigen in the samples. The standard and sample from each process stage were diluted serially and added to the reaction plate. After incubation, they were combined with enzyme-labelled antibodies against rabies virus glycoprotein, followed by the addition of chromogenic substrate solution and stop solution. The absorbance was read at a wavelength of 450 nm using a microplate reader. A standard curve was plotted with the dilution of the standard on the horizontal axis and the average absorbance of the standard on the vertical axis. The curve was fitted using a four-parameter parallel lines method to calculate the relative content of the rabies virus glycoprotein antigen in the sample.

### 2.4. Evaluation of Process Stability

Seven continuous batches of human rabies vaccine were produced according to the established process. The intermediate virus gel filtration chromatography-purified solution was tested for residual HCD amount, residual HCD fragment distribution, and glycoprotein antigen content, and dynamic trend analysis was conducted on residual HCD and antigen content. The dynamic mean and dynamic standard deviation (SD) of each batch were calculated. The dynamic mean ± 2 dynamic SD was set as the warning limit and the dynamic mean ± 3 dynamic SD was set as the action limit.

## 3. Results

### 3.1. Detection of Residual HCD in the Process Intermediates

As shown in Table 1, the detection results of the two batches were highly consistent. The HCD removal effectiveness of different processes was consistent between batches, indicating the stability of the production process. The ultrafiltration concentration, ion-exchange chromatography, and virus inactivation processes all efficiently removed HCD. The removal rate by ion-exchange chromatography was not lower than 99.9%, while the ability of gel chromatography to remove HCD was limited. After the virus harvest solution underwent ultrafiltration, ion-exchange chromatography purification, virus inactivation, and gel chromatography, the total removal rate of HCD could reach 99.99%. After conversion to the finished product, the residual HCD level was far below the approved quality standard of 2 ng/day.

### 3.2. Detection of Residual HCD Fragment Size Distribution in the Process Intermediates

**Figure 2:**
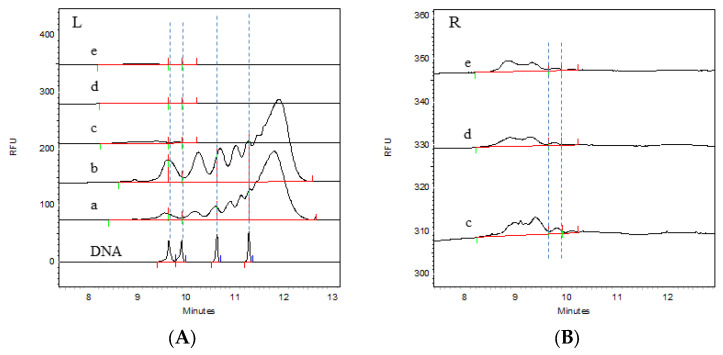
Electrophoretic profiles of the distribution of residual HCD fragments from Vero cells in each process intermediate sample of batch 202304, as determined by the CGE method. (**A**): DNA ladder 100, 200, 500, and 1000 bp; a: virus harvesting solution; b: virus ultrafiltration concentrate solution; c: virus ion-exchange chromatography solution; d: virus inactivation solution; e: virus gel filtration chromatography solution; (**B**): enlarged views of c, d, and e. The dashed lines divide fragments with different lengths.

**Figure 3:**
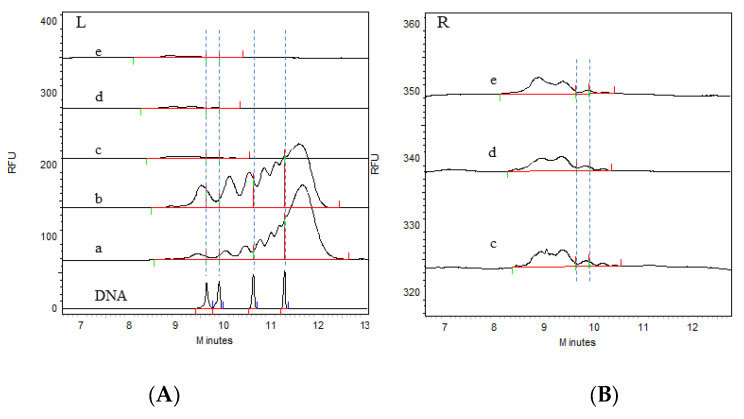
Electrophoretic profiles of the size distribution of residual HCD fragments from Vero cells in each process intermediate sample of batch 202305 as determined by the CGE method. (**A**): DNA ladder 100, 200, 500, and 1000 bp; a: virus harvesting solution; b: virus ultrafiltration concentrate solution; c: virus ion-exchange chromatography solution; d: virus inactivation solution; e: virus gel filtration chromatography solution; (**B**): enlarged views of c, d, and e. The dashed lines divide fragments with different lengths.

The CGE detection results are shown in Figure 2 and Figure 3. It can be clearly seen from the electrophoretogram that the HCD size distribution results of the two batches were highly consistent at different process stages. A comparison of the DNA markers clearly revealed that there were fragments significantly larger than 500 bp in the virus harvest solution and the virus ultrafiltration concentrate solution; moreover, the peak response value in the virus ultrafiltration concentrate solution was greater than that in the virus harvest solution, indicating that larger HCD fragments were also present in the ultrafiltration concentrate. After the ion-exchange chromatography step, the HCD size distribution was significantly reduced. The enlarged views on the right side of Figure 2 and Figure 3 show that the main size range of fragments was less than 200 bp. After the integration of electrophoretic profiles using software, the contents of the different fragments were determined, with the results shown in Table 2. It can be seen that the contents of the different fragments in the two batches were also highly consistent, and that the residual HCD fragment sizes in different process stages were consistent between batches, indicating stability in the production process. Both the virus harvesting solution and the virus ultrafiltration concentrate solution contained large amounts of residual HCD fragments, with the fragment distribution dominated by fragments larger than 1 kb. After ultrafiltration concentration, the amount of residual HCD fragments smaller than 500 bp significantly increased. After purification by ion-exchange chromatography, the total amount of residual HCD was significantly reduced, almost all large HCD fragments were removed, and the fragment size distribution was concentrated within 100 bp. After the inactivation and gel chromatography processes, the amount of HCD fragments smaller than 100 bp increased, the amount of fragments smaller than 200 bp was not lower than 95%, and fragments larger than 500 bp were not detected. Therefore, ion-exchange chromatography was the main step for removing HCD, whereas gel chromatography had a limited effect on HCD removal.

The HCD distribution of two batches of samples was detected by the CGE method. The electrophoretogram of batch 1 (202304) is shown in Figure 2, Figure 2A is the HCD distribution of the DNA ladder and different purification stages of rabies vaccine, and Figure 2B is the enlarged electrophoretogram of viral ion exchange chromatography solution, viral inactivation solution, and viral gel filtration chromatography solution. The results of the content distribution of different HCD fragments are shown in Table 2. By comparing the DNA ladder, we can clearly distinguish the distribution of HCD. The distribution of HCD in the virus harvest solution and the virus concentrate was larger than 500 bp or even 1000 bp, and the response of 100–1000 bp fragments in the virus ultrafiltration concentrate was higher than that in the virus harvest solution, indicating that HCD would also be enriched during the process of virus ultrafiltration. The HCD in the virus ion-exchange chromatography solution, virus inactivated solution, and virus gel filtration chromatography solution was mainly distributed within 100 bp, and the distribution range and content of HCD fragments were significantly reduced after the virus ion-exchange chromatography process, indicating that ion-exchange chromatography was the most effective step in removing HCD in the whole process system. The distribution content of HCD in each stage can be accurately quantified by software integration. After five processes, the HCD content of samples greater than 200 bp was less than 5%. Batch 2 (202305) also showed the same trend. The electrophoretogram is shown in Figure 3, and the distribution results of different fragments are shown in Table 2.

### 3.3. Detection of Rabies Virus Glycoprotein Antigen Content in the Process Intermediates

As shown in Table 3, the ELISA results of the two batches were highly consistent. Each process reduced the rabies virus glycoprotein antigen content to different degrees, and the degree of loss was consistent between batches, demonstrating that stability of the production process. Although the virus ultrafiltration concentration and ion-exchange chromatography processes reduced the antigen content, the antigen harvesting process ensured that the antigen content met the requirements of the finished vaccines on a commercial scale.

### 3.4. Process Stability

**Figure 4:**
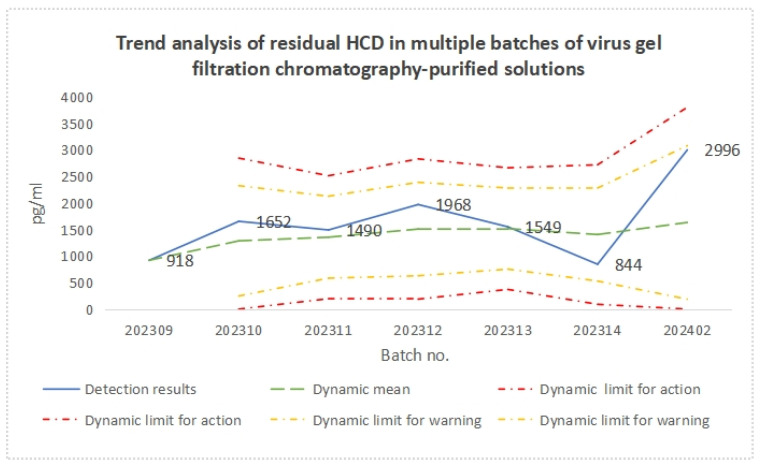
Trend analysis of residual HCD content in multiple batches of virus gel filtration chromatography-purified solutions.

The detection results and trend analysis of residual HCD in multiple batches of virus gel filtration chromatography-purified solutions are presented in Table 4 and Figure 4, respectively. The results show that, although the residual HCD level of the virus gel filtration chromatography-purified solution fluctuated among different batches, the detection results of all batches were within the dynamic warning limit, and, after conversion, the detection results of all batches met the approved quality standard (<2 ng/dose).

**Figure 5:**
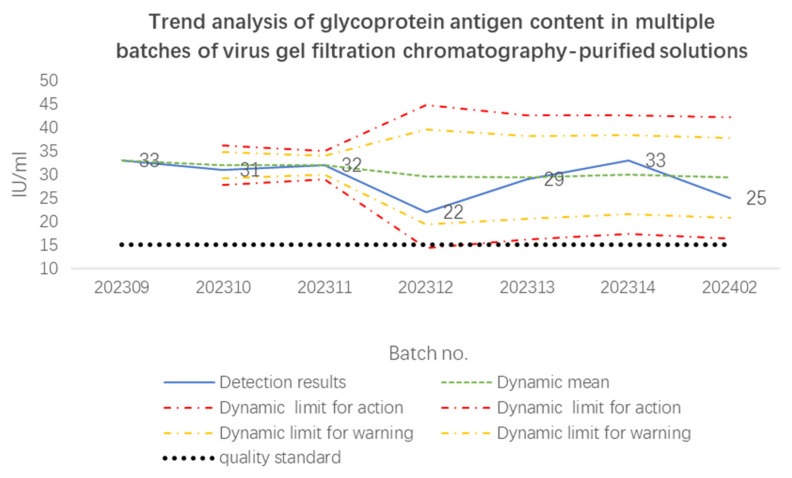
Trend analysis of glycoprotein antigen content in multiple batches of virus gel filtration chromatography-purified solutions.

Table 5 and Figure 5 present the detection results of glycoprotein antigen content in multiple batches of virus gel filtration chromatography-purified solutions. Although the glycoprotein antigen content fluctuated between batches, the detection results of all batches were within the dynamic warning limits, and the detection results of all batches far exceeded the approved quality standard (15 IU/mL).

**Figure 6:**
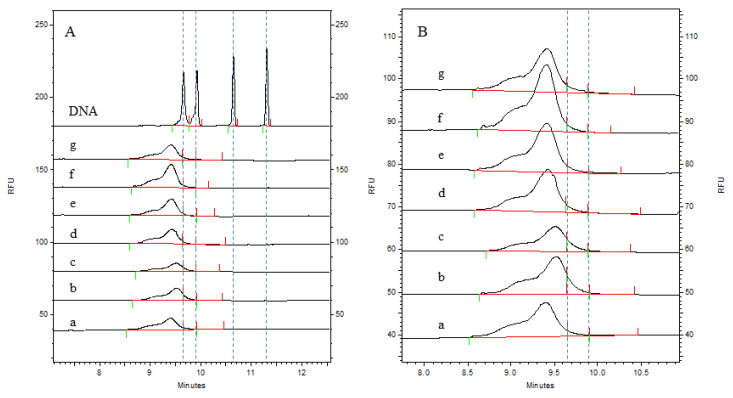
The electrophoretic profiles of residual HCD fragment size distribution in multiple batches of virus gel filtration chromatography-purified solutions, determined using the CGE method. (**A**): DNA ladder 100, 200, 500, and 1000 bp, where a to g represent the detection results of different batches of virus gel chromatography-purified solutions; (**B**): enlarged views of a to g. The dashed lines divide fragments with different lengths.

Figure 6 and Table 6 show the detection results of the residual HCD fragment size distribution in multiple batches of virus gel filtration chromatography-purified solutions. The results revealed that the size distribution of residual HCD fragments in each batch of virus gel filtration chromatography-purified solution was consistent. Due to the sequential injection of the ladder and each sample, the retention time of the characteristic peaks for each sample fluctuated slightly, and differences in the concentration of residual HCD fragments within 200 bp were detected across samples. Nevertheless, the concentrations of each sample were no lower than 98%, and no fragments larger than 500 bp were detected, demonstrating that the process is stable and suitable for large-scale batch production.

## 4. Discussion

Once rabies symptoms appear, there is no treatment option available, and the outcome is almost always fatal. Therefore, post-exposure rabies vaccination is a crucial preventive measure. Vero cells are widely used in the production of viral vaccines due to their suitability for large-scale culture and sensitivity to various virus strains [15,16]. Currently, the Vero cell rabies vaccine is the most popular human rabies vaccine in China, and this vaccine is produced from Vero cell lines, which were used to produce approximately 89% of all vaccines in 2023. Currently, the vaccine strains approved in China for use in the production of Vero cell rabies vaccines include the aG, CTN-1V, PV, and PM strains [17]. Among them, the CTN strain was isolated from a deceased rabies patient in Shandong, China, in 1956. After passage and adaptation to Vero cells, it was transformed into a fixed strain with suitable safety and immunogenicity [18]. It is the only virus strain isolated in China that is approved by the WHO for vaccine production and has independent intellectual property rights [19]. This strain is highly homologous to the street strains isolated in most regions of China. Li et al. used the CTN-1 strain to develop vaccines against various representative rabies virus strains from clades I-VII in China [20], and longitudinal clinical data revealed that the vaccine is safe and effective, making it suitable for rabies prevention and control in China [18,21,22,23].

The production process of the Vero cell rabies vaccine described in this study involved the inoculation of Vero cells with fixed rabies virus, followed by cultivation, harvesting, concentration, ion-exchange chromatography purification, virus inactivation, gel chromatography purification, and, finally, lyophilization by adding suitable stabilizers. The safety and efficacy of vaccine products are key measures of vaccine quality. Due to the complexity of rabies vaccine production processes and the numerous variables involved, removing impurities as much as possible while ensuring the effective recovery of viral antigens has always been a key focus of rabies vaccine production process research and a challenge in production process control. The final vaccine products contain large amounts of protective agents such as sucrose and human serum albumin, which pose great challenges to existing nucleic acid extraction techniques. Currently, there is no scientific and effective method for nucleic acid extraction. To ensure the scientific validity and accuracy of this study, based on the described production process, after the gel chromatography purification process, the purified solution was diluted several times according to a specific amount of antigen to produce the final product. Therefore, after gel chromatography purification, there is no possibility of adding or removing external HCD, meaning that the gel chromatography solution can effectively represent the residual HCD level in the final vaccine product, and the results showed a proportional relationship.

Residual HCD from high-passage Vero cells has potential tumorigenicity. Although the cell passages used in vaccine production are within the safe range (not exceeding 150 passages), the residual HCD components in vaccine products are complex and lack identification methods, making the purification method for the effective and stable removal of HCD still a key challenge in vaccine production research [24]. Currently, gel chromatography is a common purification method in human rabies vaccine production, but it has limited efficiency in DNA removal [25]. Previous research has confirmed that ion-exchange chromatography is a promising purification method. It purifies vaccine process products using physical methods without adding exogenous products. For example, Yang et al. used anion-exchange chromatography to purify influenza virus [26], and Shaddeau et al. used tandem ion-exchange and size-exclusion chromatography to separate vaccine components from host cell proteins and other components in cell media to detect vaccine titres [27]. In addition, some researchers have studied the use of nuclease DNA digestion and the addition of a purification process to remove nucleases for HCD removal. Although this process is effective in removing HCD, due to the lack of in-depth research on the biological activity of residual nucleases entering the human body and the low sensitivity of current nuclease content detection using ELISA [28], the accurate quantification of nucleases in the final product cannot be ensured. In addition, this process increases production costs [29,30] and carries safety uncertainties. In summary, it is appropriate to include ion-exchange chromatography in the purification process of rabies vaccines to achieve the safe and efficient removal of HCD. In this study, before virus inactivation, the virus harvest solution was first subjected to ultrafiltration and concentration to remove large particle impurities, followed by ion-exchange chromatography, which achieved a stable HCD removal rate of up to 99.99% and completely eliminated large HCD fragments that carry potential safety risks. The gel chromatography purification process after virus inactivation achieved efficient recovery of viral antigens and further removed residual HCD from the product to ensure that the concentration of residual HCD fragments smaller than 200 bp was not less than 93%. In addition, as calculated based on the dilution factor, the residual HCD level in the finished vaccines from the batches of the process stability study involved in this research was no higher than 0.72 ng/dose, which is far below the approved quality standard of 2 ng/dose.

CGE is an effective method for high-precision and high-sensitivity analysis of HCD fragment size distribution [31]. CGE analysis of nucleic acids was performed using UV or laser-induced fluorescence (LIF) detectors in combination with SYBR Gold asymmetric cyanine dye. After binding to double-stranded DNA, this dye has a high quantum yield and can enhance the fluorescence intensity by more than 1000 times [32]. Furthermore, this type of asymmetric cyanine dye has a relatively high affinity for DNA, and in CGE-LIF analysis, it can be directly added to the gel buffer to bind to the running DNA sample without special sample pretreatment. Therefore, this method has the characteristics of high sensitivity and the ability to objectively and accurately reflect the nucleic acid profile of complex samples, and thus it has been increasingly applied in the nucleic acid analysis of complex samples. There have been reports on the analysis of particle size distribution of residual HCD fragments in lentiviral products from different processes [33]. The CGE technique has also been used for the isolation of nucleic acids of different lengths or different conformations in complex samples, such as the analysis of the purity of plasmid supercoiled conformations as well as the analysis of the nucleic acid integrity and PolyA tail distribution of mRNA vaccines, and it has also assisted in completing the human genome sequencing project [34]. In the field of traditional inactivated vaccines, among the reported analyses of residual HCD distribution in vaccine products using CGE, Shen et al. introduced and validated the CGE-LIF method for analyzing the size distribution of HCD in influenza viruses cultured in MDCK cells and confirmed that the detection limit of the method for nucleic acids can be lower than 1 ng/mL [31]. The results of the present study showed that the CGE technique can be used to preliminarily evaluate the size distribution of residual HCD fragments in various rabies vaccine process intermediates. However, because this method involves the sequential injection of a single needle capillary, the retention time of the HCD characteristic peak of each sample fluctuates slightly. After subsequent standardization of the method, it can be used as a routine assay method to determine the size distribution of residual HCD fragments in purified solution.

In recent years, qPCR has generally been considered one of the most practical methods for routine HCD content determination because of its sequence specificity, sensitivity, and rapid and high-throughput nature [35,36]. The core of this detection method is the quantitative analysis of the validated specific template and the construction of the corresponding standard curve. There is a highly repetitive satellite sequence, AGMr(HindIII)-1, in Vero cells, with a monomer length of 172 bp, accounting for approximately 15% of the total genome. This sequence mainly appears in the form of long tandem repeats and is interspersed with unrelated sequences. The former has a maximum observed number of 29 long tandem repeats; the latter accounts for approximately 37% of the total genome [37]. This fragment is the unique sequence of the Vero cell genome, and the fragment distribution of this sequence and its derivatives is broad-spectrum representative of Vero cells and can be used for the quantitative analysis of HCD [38,39]. In accordance with the current edition of the Chinese Pharmacopeia, the detection of residual Vero cell DNA in bulk human rabies vaccines is conducted using the qPCR method. A standard curve is drawn using Vero cell genomic standards with OD260/OD280 values, and a quantitative analysis is performed on the 154 bp sequence of AGMr(HindIII)-1 in the HCD of the test product, with the quality standard being no higher than 3 ng/dose. Based on the FDA’s recommendation that the residual HCD should not be greater than 200 bp, the quantitative amplification of the 154 bp target fragment produces amplification products of the monomeric AGMr(HindIII)-1 sequence with no less than 154 bp and a long tandem repeat sequence, providing scientific validity to its safety evaluation. Moreover, since this detection method cannot detect sequences smaller than 154 bp or non-AGMr(HindIII)-1Vero cell genome sequences, the CGE method serves as a necessary supplement for directly observing HCD sequences.

In summary, in the production process of human rabies vaccines, ion-exchange chromatography is a key step for removing residual HCD. The production process described in this study can achieve the effective recovery of viral antigens and the efficient removal of residual HCD, and the process is stable and controllable, allowing the continuous and stable production of high-quality vaccines with excellent safety and efficacy. In addition, this study provides theoretical guidance for the optimization of the vaccine production process.

## Figures and Tables

**Table 1:** Residual HCD content Via qPCR in intermediate control samples.

Sample Name	Batch Number	Nucleic Acid Residues (pg/mL)	Total Amount of Nucleic Acid (µg)	Nucleic Acid Removal Rate	Total Removal Rate %
Virus harvest solution	202304	9.72 × 10^6^	2.11 × 10^7^	/	99.99%
Virus ultrafiltration concentrate solution		1.23 × 10^7^	3.23 × 10^6^	84.69%	
Virus ion-exchange chromatography		1.48 × 10^4^	3.07 × 10^2^	99.99%	
Virus inactivation solution		6.17 × 10^3^	1.25 × 10^2^	59.17%	
Virus gel chromatography solution		4.27 × 10^3^	1.19 × 10^2^	4.75%	
Virus harvest solution	202305	8.31 × 10^6^	1.77 × 10^7^	/	99.99%
Virus ultrafiltration concentrate solution		2.62 × 10^6^	6.84 × 10^5^	96.14%	
Virus ion-exchange chromatography solution		3.30 × 10^4^	6.80 × 10^2^	99.90%	
Virus inactivation solution		8.17 × 10^3^	1.64 × 10^2^	75.84%	
Virus gel chromatography solution		5.37 × 10^3^	1.50 × 10^2^	8.53%	

Note: Nucleic acid removal rate = 1 − (residual nucleic acid after the process/residual nucleic acid before the process) × 100%; “/” indicates not detected.

**Table 2:** Residual HCD fragment size distribution in intermediate control samples.

Sample Name	Batch Number	Proportions of Residual HCD Fragments Sorted by Size (CA%)				
		<100 bp	100–200 bp	200–500 bp	500–1000 bp	>1000 bp
Virus harvest solution	202304	4.15	1.77	8.41	17.77	67.9
Virus ultrafiltration concentrate solution		5.56	4.61	13.18	20.52	56.13
Virus ion-exchange chromatography solution		89.5	7.69	2.81	/	/
Virus inactivation solution		90.32	7.07	2.6	/	/
Virus gel chromatography solution		91.62	6.03	2.35	/	/
Virus harvest solution	202305	3.93	0.92	8.84	22.03	64.27
Virus ultrafiltration concentrate solution		8.77	3.39	20.23	27.27	40.34
Virus ion-exchange chromatography solution		85.54	7.27	7.19	/	/
Virus inactivation solution		87.38	8.13	4.49	/	/
Virus gel chromatography solution		90.93	4.74	4.33	/	/

Note: “/” indicates not detected.

**Table 3:** Antigen content in process intermediates.

Sample Name	Batch Number	Antigen Content (IU/mL)	Total Antigen (IU)	Antigen Yield %	Total Yield %
Virus harvest solution	202304	4	8.70 × 10^6^	/	8.68%
Virus ultrafiltration concentrate solution		14	3.67 × 10^6^	42.22%	
Virus ion-exchange chromatography solution		45	9.34 × 10^5^	25.44%	
Virus inactivation solution		44	8.94 × 10^5^	95.71%	
Virus gel chromatography solution		27	7.55 × 10^5^	84.47%	
Virus harvest solution	202305	4	8.53 × 10^6^	/	8.53%
Virus ultrafiltration concentrate solution		14	3.66 × 10^6^	42.90%	
Virus ion-exchange chromatography solution		44	9.08 × 10^5^	24.80%	
Virus inactivation solution		47	9.46 × 10^5^	104.18%	
Virus gel chromatography solution		26	7.27 × 10^5^	76.93%	

Note: Total antigen yield = (total antigen in the rabies virus gel chromatography purification solution)/(total antigen in the rabies virus harvest solution); “/” indicates not detected.

**Table 4:** Detection results and trend analysis of residual HCD contents in multiple batches of virus gel filtration chromatography-purified solutions.

Batch Number	HCD Residual Amount						
	Detection Results (pg/mL)	Trend Analysis					
		Dynamic Mean	Dynamic SD	Dynamic Lower Limit for Action	Dynamic Upper Limit for Action	Dynamic Lower Limit for Warning	Dynamic Upper Limit for Warning
202309	918	918	/	/	/	/	/
202310	1652	1285	519	0	2842	247	2323
202311	1490	1353	386	196	2510	582	2125
202312	1968	1507	440	187	2827	627	2387
202313	1549	1515	381	371	2660	752	2278
202314	844	1404	438	90	2717	528	2279
202402	2996	1631	722	0	3798	186	3076

Note: “/” indicates not detected.

**Table 5:** Detection results and trend analysis of glycoprotein antigen content in multiple batches of virus gel filtration chromatography-purified solutions.

Batch Number	Glycoprotein Antigen Content						
	Detection Results (IU/mL)	Trend Analysis					
		Dynamic Mean	Dynamic SD	Dynamic Lower Limit for Action	Dynamic Upper Limit for Action	Dynamic Lower Limit for Warning	Dynamic Upper Limit for Warning
202309	33	33	/	/	/	/	/
202310	31	32	1	28	36	29	35
202311	32	32	1	29	35	30	34
202312	22	30	5	14	45	19	40
202313	29	29	4	16	43	21	38
202314	33	30	4	17	43	22	38
202402	25	29	4	16	42	21	38

Note: “/” indicates not detected.

**Table 6:** Size distribution of residual HCD fragments in seven batches of process intermediates and virus gel filtration chromatography-purified solutions.

Batch Number	Residual HCD Fragment Content Sorted by Size (CA%)			
	<100 bp	100–200 bp	200 bp–500 bp	>500 bp
202309(a)	95.56	3.50	0.94	/
202310(b)	89.84	8.77	1.40	/
202311(c)	89.78	8.63	1.59	/
202312(d)	95.48	4.13	0.39	/
202313(e)	97.04	2.71	0.25	/
202314(f)	97.47	2.41	0.11	/
202402(g)	94.73	4.06	1.22	/

Note: “/” indicates not detected.

## Data Availability

The data presented in this study are available on request from the corresponding author.
